# The usefulness of cholangioscopy in the diagnosis of peribiliary cysts: a case report

**DOI:** 10.1055/a-2499-7279

**Published:** 2025-01-21

**Authors:** Juan Pablo Valencia Quivano, Juan Antonio Trejos Naranjo, Ciro Andrés Murcia Cardona, Andrés Cárdenas, Geovanny Hernández Cely

**Affiliations:** 1 25807 School of Medicine and Health Sciences, Gastroenterology Program, Universidad del Rosario, Bogotá, Colombia; 2 42705 Department of Gastroenterology & Hepatology, Fundación Cardioinfantil Instituto de Cardiología, Bogotá, Colombia; 3 28021 School of Medicine and Health Sciences, Gastroenterology Program, Universidad Nacional de Colombia, Bogota, Colombia; 4 16493 Institut de Malalties Digestives i Metabòliques, Hospital Clinic de Barcelona, Barcelona, Spain

A 63-year-old woman with no medical or surgical history came to the outpatient clinic presenting with 6 months of unquantified unintentional weight loss and jaundice. Physical examination documented only jaundice with no other alterations.

Laboratory results documented an obstructive biliary pattern ( Table 1 ). Magnetic resonance imaging (MRI) showed no evidence of neoplasia and a dilatation of the choledochus and intraductal image ( Fig. 1 ). Echo-endoscopy showed only an image suggestive of a 2-mm stone in the choledochus ( Fig. 2 ). Endoscopic retrograde cholangiopancreatography and cholangioscopy was then performed using direct visualization by SpyGlass (Boston Scientific, Marlborough, Massachusetts, USA) in which multiple subepithelial, rounded and translucent lesions corresponding to cysts were observed, some of which ruptured spontaneously during the procedure ( Fig. 3 , Video 1 ). Jaundice resolved after the procedure. Biopsies of the lesions were taken by SpyBite (Boston Scientific) without finding neoplastic pathology. After 6 months of observation, the patient is asymptomatic and continues to be followed up.

**Table:** **Table 1** Laboratory results

Laboratory	28/02/23	Reference values
Leukocytes (×10^3)	6220	4.5–11.3
Hemoglobin (g/dL)	14.8	12.3–15.3
Platelets (×10^3)	188000	150–450
Alkaline phosphatase (U/L)	710	35–104
Gamma glutamyl transferase (U/L)	774	6–42
Alanine amino transferase (ALT) (U/L)	89	0–31
Aspartate amino transferase (AST) (U/L)	86	0–32
Total bilirubin (mg/dL)	2.55	0–1
Direct bilirubin (conjugated) (mg/dL)	2.1	0–0.3
Creatinine (mg/dL)	0.83	0.51–0.95
INR	0.85	–
CA 19–9	52	0–39
ACE	2.5	0–4.3

**Fig. 1:**
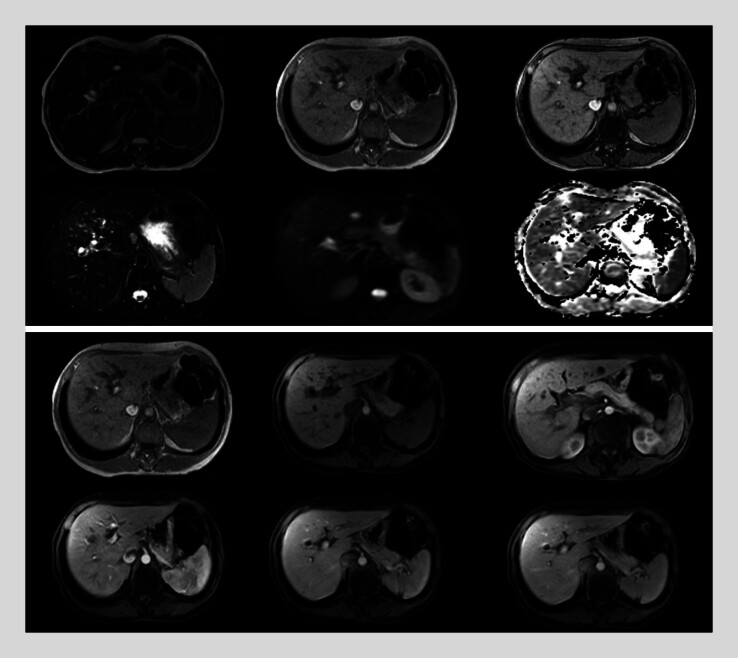
Dilatation of the choledochus with an image that could correspond to calculus (only visualized in a sequence). However, there is an abrupt termination of the choledochus and saccular dilatations of the intrahepatic biliary tract, so it is not possible to rule out periampullary lesions. Endoscopic evaluation is suggested. The small cystic lesion of the body of the pancreatic head could correspond to intraductal papillary mucinous intraductal neoplasm with no ominous signs. Cysts are simple hepatic cysts.

**Fig. 2:**
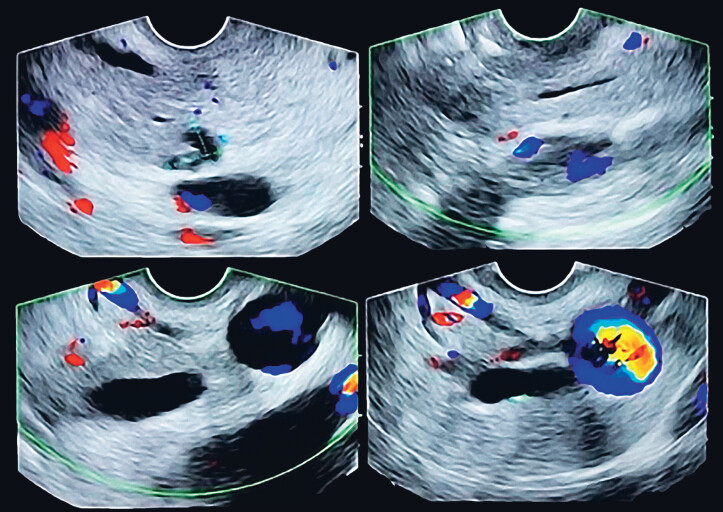
Pancreatic head cyst measuring 6×9 mm, without communication with the main or secondary pancreatic duct. 7 mm choledochus with micro calculus of 2 mm inside. Papilla are endoscopically and endosonographically normal.

**Fig. 3:**
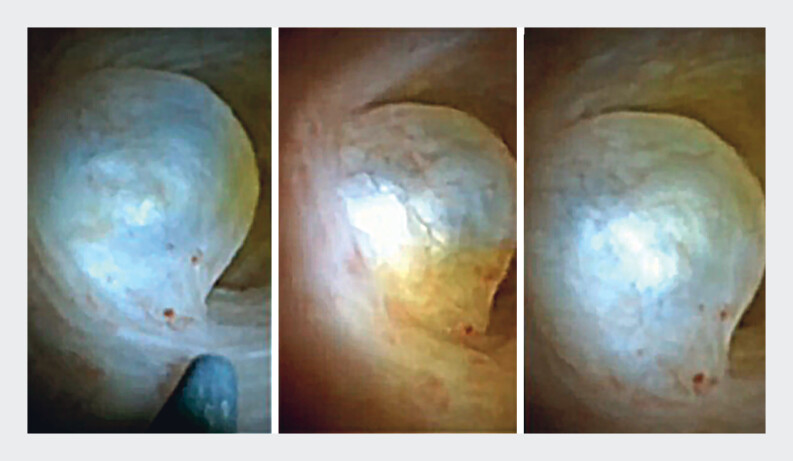
Cholangiopancreatography **:** Multiple filling defects at the level of the middle choledochus, proximal choledochus, right and left hepatic duct, with dilated intra- and extra-hepatic biliary tract. Cholangioscopy **:** Multiple subepithelial, rounded, translucent lesions corresponding to cysts, some of which rupture spontaneously. At the level of the left hepatic duct, there is a cyst that generates partial occlusion of two ducts that is broken by SpyBite forceps.

Endoscopic retrograde cholangiopancreatography and cholangioscopy was performed using direct visualization. Multiple subepithelial, rounded, and translucent lesions were observed corresponding to cysts, some of which ruptured spontaneously during the procedure.Video 1

Peribiliary cysts 1 are saccular structures formed from the dilatation of peribiliary glands. They are usually tiny (<10 mm) and do not communicate with the bile ducts 2 . They are mainly associated with cirrhosis and enolism (38%). They are more prevalent in the male sex (80%), with less female representation. Regarding clinical manifestations, obstructive and constitutional biliary syndrome with consequent suspicion of neoplastic obstruction of the biliary tract is the reason for consultation and clinical approach in 19% of patients 3 **.** With respect to detection, although computed tomography (CT) and MRI can document typical cystic lesions and thus be diagnostic in up to 48% and 64% of cases, respectively, in some scenarios cholangioscopy may be required to characterize and biopsy in order to clarify the diagnosis 4 **.** Regarding treatment, the authors agree that asymptomatic patients do not require specific management or follow-up. In our case, cholangioscopy was indicated due to obstructive biliary involvement, with subsequent resolution of symptoms.

Endoscopy_UCTN_Code_TTT_1AQ_2AK

## Notes

Endoscopy E-Videos https://eref.thieme.de/e-videos
*E-Videos* is an open access online section of the journal *Endoscopy* , reporting on interesting cases and new techniques in gastroenterological endoscopy. All papers include a high-quality video and are published with a Creative Commons CC-BY license. Endoscopy E-Videos qualify for HINARI discounts and waivers and eligibility is automatically checked during the submission process. We grant 100% waivers to articles whose corresponding authors are based in Group A countries and 50% waivers to those who are based in Group B countries as classified by Research4Life (see: https://www.research4life.org/access/eligibility/ ). This section has its own submission website at https://mc.manuscriptcentral.com/e-videos .
